# Mass conservative reaction–diffusion systems describing cell polarity

**DOI:** 10.1002/mma.7162

**Published:** 2021-01-18

**Authors:** Evangelos Latos, Takashi Suzuki

**Affiliations:** ^1^ Institut für Mathematik und Wissenschaftliches Rechnen University of Graz Heinrichstraße 36 Graz 8010 Austria; ^2^ Center for Mathematical Modeling and Data Science Osaka University Osaka Japan

**Keywords:** Chemical reaction–diffusion system, Fix–Caginalp equation, Global dynamics, Total mass conservation system

## Abstract

A reaction–diffusion system with mass conservation modeling cell polarity is considered. A range of the parameters is found where the *ω*‐limit set of the solution is spatially homogeneous, containing constant stationary solution as well as possible nontrivial spatially homogeneous orbit.

## 1 INTRODUCTION

The present work studies the following mass‐conserved reaction–diffusion system:

(1) $$\begin{array}{ccc} & u_{t}=D\Delta u+f(u,v), & \\ & \tau v_{t}=\Delta v-f(u,v),\;\;\text{in}\;\Omega \times (0,T),\\ & \frac{\partial }{\partial \nu }(u,v)=0,\;\;\;\;\;\;\text{on}\;\partial \Omega \times (0,T),\\ & {\left.\left(u,v\right)\right|}_{t=0}=(u_{0}(x),v_{0}(x)),\;\text{in}\;\Omega , \end{array}$$

where Ω ⊂ **R**^*N*^ is a bounded domain with smooth boundary *∂*Ω, *ν* is the outer unit normal vector, *D*, *τ* are positive constants, and $(u_{0},v_{0})=(u_{0}(x),v_{0}(x))\geq 0$, (*u*_0_, *v*_0_) ≢ 0, are the nonnegative, nontrivial initial values, taken to be sufficiently smooth.

Given the sufficiently smooth nonlinearity *f* = *f*(*u*, *v*), standard theory allows the existence of a unique local‐in‐time classical solution (u,v)=(u(·,t),v(·,t)) to (1), as it can be seen in other studies,^1^, ^2^, ^3^, ^4^, ^5^, ^6^, ^7^ we also refer the interested reader to Pierre's monograph^8^ for a more complete presentation of the history of such systems. The solution (*u*, *v*) has the following total mass conservation property:

(2) $$\frac{d}{dt}\int_{\Omega }u+\tau v\;dx=0.$$

The class of models that we are going to study, were proposed in Otsuji et al.^9^ to describe cell polarity. The proposed mechanism shall separate different species inside the cell according to their diffusion coefficients, that is, slow and fast diffusions shall localize the species near the membrane and in the cytosol, respectively. Three kinds of molecules are interacting. Each one of them has two phases, active and inactive, which are characterized by slow and fast diffusions, respectively. The model problem (1) focuses on these two phases of a single species, ignoring the interactions between the other species.

The model shall allow Turing pattern,^10^ which is the appearance of spatially inhomogeneous stable stationary states induced by diffusion. In their study, Otsuji et al.^9^ suggest the following three models for this purpose:

(3) $$\begin{array}{ccc} & f(u,v)=-\frac{au}{u^{2}+b}+v, & \\ & f(u,v)=-\alpha_{1}\left[\frac{u+v}{{\left(\alpha_{2}(u+v)+1\right)}^{2}}-v\right],\\ & f(u,v)=\alpha_{1}(u+v)[(\alpha u+v)(u+v)-\alpha_{2}], \end{array}$$

where *a*, *b*, *α*, *α*_1_, and *α*_2_ are positive constants.

In their study, Mori et al.^11^ suggested

(4) $$f(u,v)=b_{1}v\left[\frac{\gamma u^{2}}{k^{2}+u^{2}}+k_{0}\right]-\delta u,$$

where *b*_1_, *γ*, *δ*, *k*_0_, *k* are positive constants (see Mori et al.^11^). System (1) with the above reaction term will be referred in the following as the fourth model and is the main topic of study in this paper. The results of this paper can be directly applied to more general reaction terms of the type

$$f(u,v)=b_{1}v\left[\frac{\gamma u^{m}}{k^{2}+u^{m}}+k_{0}\right]-\delta u$$

with *m* > 2.

The main characteristics of the system are the following:

- Quasi positivity for $\left(\begin{array}{c} f(u,v)\\ -f(u,v) \end{array}\right)$ provides positivity for (*u*, *v*):
  $$f=f(u,v):{\bar{\mathbb{R}}}_{+}^{2}\to \mathbb{R},\;\text{locally Lipschitz continuous with}\;f(0,v)\geq 0\geq f(u,0).$$
  Therefore, the solution is nonnegative, provided that nonnegative initial data are given.
- Mass conservative reaction–diffusion system: $\frac{d}{dt}\int_{\Omega }u+\tau v\;dx=0\Rightarrow$
  (5) $$\frac{1}{\left|\Omega \right|}\int_{\Omega }u+\tau v\;dx=\lambda =\frac{1}{\left|\Omega \right|}\int_{\Omega }u_{0}+\tau v_{0}\;dx.$$

For the global existence and uniform‐in‐time bounds of nonnegative classical solutions to this system in all space dimension, we refer the interested reader to Theorem 1.1 in Fellner et al.^12^ Actually, Fellner et al.^12^ consider an even more general class of systems where the reaction terms might have a (slightly super‐) quadratic growth.

In this paper, we give an answer to the natural question which rises next about the asymptotic behavior of the solution and whether it converges to the equilibrium. Namely, is the solution to this fourth model (4) asymptotically spatially homogeneous or do we have a Turing paradigm (stable nonconstant stationary state under the local enhancement and long‐range inhibition)?

This work is organized as follows: in Section 2, we summarize what has been done in the previous relevant models. In Section 3, we present and prove some of the key features of the fourth model, and we state our main Theorem 5. In Section 4, we prove our main result.

## 2 REVIEW OF THE PREVIOUS WORK

In the first and the second models, the stationary state is described by the elliptic eigenvalue problem with nonlocal term, with the eigenvalue associated with the total mass that is conserved in time. The stationary state has a variational functional *J*, while there is a Lyapunov functional *L*(*u*, *v*) for the nonstationary problem. This Lyapunov functional is reduced to the stationary variational functional, if the total mass of (*u*, *v*) is prescribed. This remarkable structure, called semi‐unfolding minimality, induces dynamical stability of the local minimizer of *J*. We will briefly revisit what has already been done for these models.

### 2.1 First model

If we let

$$f(u,v)=h(u)+kv,\;h(u)=-\frac{au}{u^{2}+b},\;k=1,$$

the first model takes the form

(6) $$\begin{array}{ccc} & u_{t}=D\Delta u+h(u)+kv, & \\ & \tau v_{t}=\Delta v-h(u)-kv,\;\;\;\text{in}\;\Omega \times (0,T),\\ & \frac{\partial }{\partial \nu }(u,v)=0,\;\;\;\;\;\text{on}\;\partial \Omega \times (0,T),\\ & {\left.\left(u,v\right)\right|}_{t=0}=(u_{0}(x),v_{0}(x)),\;\text{in}\;\Omega . \end{array}$$

Henceforth, *C*_*i*_, i=1,2,…,9 denote positive constants independent of *t*. Since this *h* = *h*(*u*) is a smooth function of *u* ∈ **R** satisfying

(7) $$h(0)=0\geq h(u)\geq -C_{1},\;u\geq 0,$$

if $0\leq (u_{0},v_{0})=(u_{0}(x),v_{0}(x))\in C^{2}{\left(\bar{\Omega }\right)}^{2}$, for simplicity, from here on, we denote $X=C^{2}{\left(\bar{\Omega }\right)}^{2}$, then problem (6) admits a unique classical solution (u,v)=(u(·,t),v(·,t)) uniformly bounded, and global‐in‐time (Theorem 1.1 in Fellner et al.^12^). Therefore, the orbit $O={\left\{(u(\cdot ,t),v(\cdot ,t))\right\}}_{t\geq 0}$ is compact in *X*, and hence, the *ω*‐limit set defined by

(8) $$\omega (u_{0},w_{0})=\{(u_{*},w_{*})|\exists t_{k}↑+\infty s.t.\|u(\cdot ,t_{k})-u_{*}(\lambda ),w(\cdot ,t_{k})-w_{*}(\lambda ){\|}_{X}=0\}$$

is nonempty, compact, and connected.

With

w=Du+v,ξ=1−τD,

the system (6) transforms into

(9) $$\begin{array}{ccc} & u_{t}=D\Delta u+h(u)-kDu+kw, & \\ & \tau w_{t}+\xi u_{t}=\Delta w,\;\;\;\;\;\text{in}\;\Omega \times (0,T),\\ & \frac{\partial }{\partial \nu }(u,w)=0,\;\;\;\;\;\;\text{on}\;\partial \Omega \times (0,T),\\ & {\left.\left(u,w\right)\right|}_{t=0}=(u_{0}(x),w_{0}(x)),\;\;\text{in}\;\Omega \end{array}$$

for $w_{0}=Du_{0}+v_{0}$. In the stationary state, we have

$$\Delta w=0\;\text{in}\;\Omega ,\;{\left.\frac{\partial w}{\partial \nu }\right|}_{\partial \Omega }=0,$$

and therefore, this *w* is a constant denoted by $\bar{w}$. $\bar{w}$ is prescribed by the initial value using (5):

(10) $$\tau \bar{w}+\frac{\xi }{\left|\Omega \right|}\int_{\Omega }u\;dx=\lambda =\frac{1}{\left|\Omega \right|}\int_{\Omega }\tau w_{0}+\xi u_{0}\;dx.$$

We thus obtain

(11) $$-D\Delta u=q(u)+\frac{k}{\tau }\left(\lambda -\frac{\xi }{\left|\Omega \right|}\int_{\Omega }u\;dx\right),\;{\left.\frac{\partial u}{\partial \nu }\right|}_{\partial \Omega }=0$$

for

q(u)=h(u)−kDu.

The set of stationary solutions to (6), denoted by *E*_*λ*_, is thus defined in accordance with *λ* in (5), that is, (*u*, *v*) ∈ *E*_*λ*_ if and only if *u* = *u*(*x*) is a solution to (11) for ξ=1−τD, and $v=\bar{w}-Du$, where $\bar{w}$ is a constant defined by

$$\bar{w}=\frac{1}{\tau }\left(\lambda -\frac{\xi }{\left|\Omega \right|}\int_{\Omega }u\right).$$

By exploiting the above observations, Morita and Ogawa^7^ and Morita^5^ studied the spectral analysis of the stationary solution. The purpose of Latos and Suzuki^3^ was to study the previous results from the point of view of global dynamics. In fact, with the use of the Lyapunov functional, they showed the existence of a global‐in‐time solution to (6) in $X=C^{2}{\left(\bar{\Omega }\right)}^{2}$ with compact orbit. The following theorem is proven by the existence of the Lyapunov functional to (6):

$$\frac{d}{dt}\left\{\xi \int_{\Omega }\frac{D}{2}|\nabla u{|}^{2}-Q(u)\;dx+\frac{\tau k}{2}\|w{\|}_{2}^{2}\right\}+\xi \|u_{t}{\|}_{2}^{2}+k\|\nabla w{\|}_{2}^{2}=0.$$

Theorem 1 (^3^) If ξ=1−τD>0, it holds that *ω*(*u*_0_, *v*_0_) ⊂ *E*_*λ*_.

Remark 1 From the result^12^ established later, the restriction on the space dimension N=1,2,3, in Latos and Suzuki,^3^ is excluded for the compactness of O. This extension is also valid to the second model described below.

The problem (11) has a variational structure. Thus, *u* = *u*(*x*) is a solution if and only if $J_{\lambda }^{\prime }(u)=0$, where

(12) $$J_{\lambda }(v)=\int_{\Omega }\frac{D}{2}|\nabla v{|}^{2}-Q(v)-\frac{k}{\tau }\lambda v\;dx-\frac{k\xi }{2\tau |\Omega |}{\left(\int_{\Omega }v\right)}^{2},\;v\in H^{1}(\Omega )$$

for *Q′*(*u*) = *q*(*u*). Then we obtain the dynamical stability of local minimizers of this functional.

Theorem 2 (^3^) Let ξ=1−τD>0 and *h* = *h*(*u*) to be a real‐analytic function in *u* ∈ **R**. Given *λ* > 0, let *u*_∗_ = *u*_∗_(*x*) ∈ *H*^1^(Ω) be a local minimizer of $J_{\lambda }=J_{\lambda }(v)$ in (12), and put

$$w_{*}=\frac{1}{\tau }\left(\lambda -\frac{\xi }{\left|\Omega \right|}\int_{\Omega }u_{*}\;dx\right).$$

Then this stationary solution (*u*_∗_, *w*_∗_) to (9), for (*u*_0_, *w*_0_) satisfying (10), is dynamically stable in *H*^1^(Ω) × *L*^2^(Ω). Thus, any *ε* > 0 admits *δ* > 0 such that if (*u*_0_, *w*_0_) ∈ *H*^1^(Ω) × *L*^2^(Ω) satisfies

$$\|u_{0}-u_{*}{\|}_{H^{1}}^{2}+\|w_{0}-w_{*}{\|}_{2}^{2}<\delta ,\;\frac{1}{\left|\Omega \right|}\int_{\Omega }\tau w_{0}+\xi u_{0}\;dx=\lambda ,$$

then it holds that

$$\underset{t\geq 0}{\sup}\left\{\|u(\cdot ,t)-u_{*}{\|}_{H^{1}}^{2}+\|w(\cdot ,t)-w_{*}{\|}_{2}^{2}\right\}<\varepsilon$$

for the solution (u,w)=(u(·,t),w(·,t)) to (9).

### 2.2 Second model

By letting

$$f(u,v)=h(u+v)+\alpha_{1}v,\;h(u+v)=-\frac{\alpha_{1}(u+v)}{{\left(\alpha_{2}(u+v)+1\right)}^{2}},$$

the model takes the form

(13) $$\begin{array}{ccc} & u_{t}=D\Delta u+h(u+v)+\alpha_{1}v, & \\ & \tau v_{t}=\Delta v-h(u+v)-\alpha_{1}v,\;\;\;\text{in}\;\Omega \times (0,T),\\ & \frac{\partial }{\partial \nu }(u,v)=0,\;\;\;\;\text{on}\;\partial \Omega \times (0,T),\\ & {\left.\left(u,v\right)\right|}_{t=0}=(u_{0}(x),v_{0}(x)),\;\;\;\;\text{in}\;\Omega . \end{array}$$

Since *h* = *h*(*z*) is a smooth function of *z* ∈ **R** satisfying

(14) $$h(0)=0\geq h(z)\geq -C_{2},\;u\geq 0,$$

if $(u_{0},v_{0})\in X=C^{2}{\left(\bar{\Omega }\right)}^{2}$ with $(u_{0},v_{0})=(u_{0}(x),v_{0}(x))\geq 0$, the problem (13) admits a uniformly bounded unique classical solution (u,v)=(u(·,t),v(·,t))≥0, global‐in‐time.

With

$$w=Du+v,\;z=u+v,\;\xi =\frac{1-\tau D}{\tau -1},\;\alpha =\frac{1-D}{\tau -1}$$

and

$$g(z)=(1-D)h(z)-\alpha_{1}Dz,$$

the system (13) transforms into

(15) $$\begin{array}{ccc} & z_{t}=D\Delta z+(w_{t}-D\Delta w+\alpha_{1}w)+g(z), & \\ & w_{t}+\xi z_{t}=\alpha \Delta w,\;\;\;\;\;\text{in}\;\Omega \times (0,T),\\ & \frac{\partial }{\partial \nu }(z,w)=0\;\;\;\;\;\;\;\text{on}\;\partial \Omega \times (0,T),\\ & {\left.\left(z,w\right)\right|}_{t=0}=(z_{0}(x),w_{0}(x)),\;\;\text{in}\;\Omega , \end{array}$$

where $\left(z_{0},w_{0})=(u_{0}+v_{0},Du_{0}+v_{0}\right)$. The orbit $O={\left\{(u(\cdot ,t),w(\cdot ,t))\right\}}_{t\geq 0}$ to (15) is thus compact in $X=C^{2}{\left(\bar{\Omega }\right)}^{2}$, and hence, the *ω*‐limit set defined by

$$\omega (u_{0},w_{0})=\{(u_{*},w_{*})|\exists t_{k}↑+\infty s.t.\|u(\cdot ,t_{k})-u_{*}(\lambda ),w(\cdot ,t_{k})-w_{*}(\lambda ){\|}_{X}=0\}$$

is nonempty, compact, and connected.

First, total mass conservation arises in the form of

(16) $$\frac{1}{\left|\Omega \right|}\int_{\Omega }\xi z+w\;dx=\lambda =\frac{1}{\left|\Omega \right|}\int_{\Omega }\xi z_{0}+w_{0}\;dx.$$

Second, there is a Lyapunov functional defined by

(17) $$L=L(z,w)=\int_{\Omega }\frac{\alpha +D}{2}|\nabla w{|}^{2}+\frac{k}{2}w^{2}+\frac{\xi D}{2}|\nabla z{|}^{2}-\xi G(z)\;dx,$$

satisfying

(18) $$\frac{dL}{dt}+\xi \|z_{t}{\|}_{2}^{2}+\|w_{t}{\|}_{2}^{2}+\alpha D\|\Delta w{\|}_{2}^{2}+\alpha k\|\nabla w{\|}_{2}^{2}=0.$$

Third, in the stationary state of (15), the component *w* = *w*(*x*) is spatially homogeneous similarly, denoted by $w=\bar{w}\in \text{R}$. Hence, it holds that

(19) $$\bar{w}=\lambda -\frac{\xi }{\left|\Omega \right|}\int_{\Omega }z\;dx$$

by (16). Plugging (19) into the first equation of (15), we see that the stationary state of (9) is reduced to a single equation concerning *z* = *z*(*x*), that is,

(20) $$-D\Delta z=g(z)+k\left(\lambda -\frac{\xi }{\left|\Omega \right|}\int_{\Omega }z\;dx\right),\;{\left.\frac{\partial z}{\partial \nu }\right|}_{\partial \Omega }=0.$$

This problem is the Euler–Lagrange equation corresponding to the variational functional

(21) $$J_{\lambda }(z)=\int_{\Omega }\frac{D}{2}|\nabla z{|}^{2}-G(z)-k\lambda z\;dx+\frac{k\xi }{2|\Omega |}{\left(\int_{\Omega }z\;dx\right)}^{2},\;z\in H^{1}(\Omega ).$$

Thus, the set of stationary solutions is associated with *λ* in (16), denoted by *E*_*λ*_. We say that $(z,\bar{w})\in E_{\lambda }$, if *z* ∈ *H*^1^(Ω) solves (20) and $\bar{w}\in \text{R}$ is defined by (19).

Then we obtain the following results similarly.

Theorem 3 (^4^) If $\xi =\frac{1-\tau D}{\tau -1}>0$, it holds that *ω*(*z*_0_, *w*_0_) ⊂ *E*_*λ*_.

Theorem 4 (^4^) Let $\xi =\frac{1-\tau D}{\tau -1}>0$ and *h* = *h*(*z*) to be a real‐analytic function in *z* ∈ **R**. Given *λ* > 0, let *z* ∗ =*z*_∗_(*x*) ∈ *H*^1^(Ω) be a local minimizer of $J_{\lambda }=J_{\lambda }(z)$ in (21), and put

$$w_{*}=\lambda -\frac{\xi }{\left|\Omega \right|}\int_{\Omega }z_{*}\;dx.$$

Then the stationary solution (*z*_∗_, *w*_∗_) to (15) is dynamically stable in *H*^1^(Ω) × *L*^2^(Ω). Thus, any *ε* > 0 admits *δ* > 0 such that if (*z*_0_, *w*_0_) ∈ *H*^1^(Ω) × *L*^2^(Ω) satisfies

$$\|z_{0}-z_{*}{\|}_{H^{1}}^{2}+\|w_{0}-w_{*}{\|}_{2}^{2}<\delta ,\;\frac{1}{\left|\Omega \right|}\int_{\Omega }w_{0}+\xi u_{0}\;dx=\lambda ,$$

then it holds that

$$\underset{t\geq 0}{\sup}\left\{\|z(\cdot ,t)-z_{*}{\|}_{H^{1}}^{2}+\|w(\cdot ,t)-w_{*}{\|}_{2}^{2}\right\}<\varepsilon$$

for the solution (z,w)=(z(·,t),w(·,t)) to (15).

Remark 2 We note the following facts. First, the local minimizer in Theorems 2 and 4 may be degenerate. Second, there is a correspondence between the Morse index of the linearized operator around the stationary solution (*u*_∗_, *z*_∗_) or (*z*_∗_, *w*_∗_) and that of *u*_∗_ or *z*_∗_ as a critical point of the variational functional *J*_*λ*_. This property is called the spectral comparison, and a result in this direction is obtained in Latos et al.^4^ for the second model.

## 3 THE MODEL AND THE RESULT

We skip the third model

$$f(u,v)=\alpha_{1}(u+v)[(\alpha u+v)(u+v)-\alpha_{2}],$$

because it does not satisfy the quasi‐positivity. Hence in this work we consider the fourth model, (1) for

(22) $$f(u,v)=vb\left[\frac{\gamma u^{2}}{k^{2}+u^{2}}+k_{0}\right]-\delta u,$$

where *δ* > 0. One can consider the more general reaction term used in Holmes and Edelstein‐Keshet,^13^

$$f(u,v)=vb\left[\frac{\gamma u^{m}}{k^{2}+u^{m}}+k_{0}\right]-\delta u,\;m>2$$

in the argument below.

Putting

(23) $$a(u)=b\left(\frac{\gamma u^{2}}{k^{2}+u^{2}}+k_{0}\right),$$

we obtain *f*(*u*, *v*) = *va*(*u*) − *δu* and

(24) $$a_{0}\equiv bk_{0}\leq a(u)\leq b(\gamma +k_{0})\equiv a_{1},\;0\leq a^{\prime }(u)\leq \alpha (k)$$

with

$$\alpha (k)=\underset{u>0}{\max}\frac{2b\gamma k^{2}u}{{\left(k^{2}+u^{2}\right)}^{2}}=\frac{3\sqrt{3}b\gamma }{16k}.$$

Therefore, this model is reduced to

(25) $$\begin{array}{ccc} & u_{t}=D\Delta u+va(u)-\delta u, & \\ & \tau v_{t}=\Delta v-va(u)+\delta u,\;\;\text{in}\;\Omega \times (0,T),\\ & \frac{\partial }{\partial \nu }(u,v)=0,\;\;\;\;\;\;\text{on}\;\partial \Omega \times (0,T).\\ & {\left.\left(u,v\right)\right|}_{t=0}=(u_{0}(x),v_{0}(x))\;\;\;\;\text{in}\;\Omega . \end{array}$$

The nonlinearity *a*(*u*) in (23) is not so wild. If it is a contact denoted by *a* > 0, the system (25) is linear, but a special form of the first model. Hence the stationary state is reduced to

(26) $$-D\Delta u=-(\delta +aD)u+\frac{a}{\tau }\left(\lambda -\frac{\xi }{\left|\Omega \right|}\int_{\Omega }u\right),\;{\left.\frac{\partial u}{\partial \nu }\right|}_{\partial \Omega }=0$$

for ξ=1−τD and

(27) $$\lambda =\frac{1}{\left|\Omega \right|}\int_{\Omega }u_{0}+\tau v_{0}\;dx.$$

There is a unique spatially homogeneous solution to (26), that is,

$$u_{*}=\frac{a\lambda }{a+\tau \delta }.$$

The linearized operator around this *u*_∗_ is given by

$$L\varphi =-D\Delta \varphi +(\delta +aD)\varphi +\frac{a\xi }{\tau |\Omega |}\int_{\Omega }\varphi \;dx,\;{\left.\frac{\partial \varphi }{\partial \nu }\right|}_{\partial \Omega }=0.$$

Using the eigenvalues and eigenfunctions of −Δ under the Neumann boundary condition, we see that this *L* is nondegenerate always. Thus there is no Turing pattern in this case, that is, in the case when *a*(*u*) is a constant.

We can actually confirm the linearized stability of this spatially homogeneous stationary solution (*u*_∗_, *v*_∗_) to (25) for *v*_∗_ satisfying *λ* = *u*_∗_ + *τv*_∗_. In fact, this linearized equation takes the form

$$\begin{array}{ccc} & \frac{\partial }{\partial t}\left(\begin{array}{c} z\\ w \end{array}\right)=\left(\begin{array}{cc} D\Delta -\delta & a\\ \tau^{-1}\delta & \tau^{-1}\Delta -\tau^{-1}a \end{array}\right)\left(\begin{array}{c} z\\ w \end{array}\right)\;\text{in}\;\Omega \times (0,T) & \\ & {\left.\frac{\partial }{\partial \nu }(z,w)\right|}_{\partial \Omega }=0,\;\frac{1}{\left|\Omega \right|}\int_{\Omega }z+\tau w\;dx=0. \end{array}$$

Using the eigenvalues and eigenfunctions of −Δ under the Neumann boundary condition again, we see that all the eigenvalues of the linearized operator are real and negative. Hence, (*u*_∗_, *v*_∗_) is asymptotically stable. In spite of these simple profiles of the solution for the case that *a*(*u*) = *a* > 0 is a constant, the global dynamics of (25) for (23) is not subject to a Lyapunov functional.

To confirm this property, we take a look at the stationary problem to (25):

(28) $$\begin{array}{ccc} & -D\Delta u+\delta u=va(u), & \\ & -\Delta v=-va(u)+\delta u\;\text{in}\;\Omega ,\\ & {\left.\frac{\partial }{\partial \nu }(u,v)\right|}_{\partial \Omega }=0, \end{array}$$

with

(29) $$\frac{1}{\left|\Omega \right|}\int_{\Omega }u+\tau v\;dx=\lambda .$$

By the argument in the previous section, the function w=Du+v in (28) is a constant denoted by $\bar{w}$, which is determined by (29):

$$\bar{w}=\frac{1}{\tau }\left(\lambda -\frac{\xi }{\left|\Omega \right|}\int_{\Omega }u\;dx\right).$$

Therefore, the system (28) is reduced to

(30) $$-D\Delta u+\left(\delta +Da(u)\right)u=\frac{a(u)}{\tau }\left(\lambda -\frac{\xi }{\left|\Omega \right|}\int_{\Omega }u\;dx\right)\;\text{in}\;\Omega ,\;{\left.\frac{\partial u}{\partial \nu }\right|}_{\partial \Omega }=0.$$

We see that (30) admits no variational functional unless *a*(*u*) is a constant as in (26). Therefore, no Lyapunov functional is expected in the nonstationary problem (25).

The first observation is the existence of a unique spatially homogeneous stationary solution to (25).

Proposition 1 For every *λ* > 0, let *G*_*λ*_ be the set of solutions $(u_{*},v_{*})\in {\text{R}}^{2}$ to

(31) $$u_{*}+\tau v_{*}=\lambda ,\;f(u_{*},v_{*})=0,$$

for *f* = *f*(*u*, *v*) defined by (22). Then *G*_*λ*_ is composed of at least one and at most three elements.

Equality (31) is equivalent to

(32) $$A(u_{*})=B(u_{*}),$$

where

$$A(u)=\frac{\tau \delta }{b}+\gamma +k_{0}+\frac{\lambda \tau \delta }{b(u-\lambda )},\;B(u)=\frac{\gamma k^{2}}{k^{2}+u^{2}}.$$

Thus, *u*_∗_ is a zero of a qubic polynomial, and the number of *G*_*λ*_ is at most three. Since

B(0)<A(0),A(λ)=−∞<B(λ),

on the other hand, there is *u*_∗_ ∈ (0, *λ*) satisfying (32). Hence, we obtain the result. □

Remark 3 There is a case that *G*_*λ*_ is composed of three elements. In the case described in Morita and Shinjo,^6^ two of them are stable, and the other is unstable, as stationary solutions to the ordinary differential equation (50) given below.

Put $\left(u_{*},v_{*})=(u_{*}(\lambda ),v_{*}(\lambda )\right)$ in Proposition 1. The linearized operator around the solution *u*_∗_ = *u*_∗_(*λ*) to (30) is given by

(33) $$L\varphi =-D\Delta \varphi +(\delta +Da(u_{*}))\varphi +Da^{\prime }(u_{*})u_{*}\varphi -\frac{a^{\prime }(u_{*})}{\tau }\left(\lambda -\frac{\xi }{\left|\Omega \right|}\int_{\Omega }u_{*}\;dx\right)\varphi +\frac{a(u_{*})\xi }{\tau |\Omega |}\int_{\Omega }\varphi \;dx\;\text{in}\;\Omega ,\;{\left.\frac{\partial \varphi }{\partial \nu }\right|}_{\partial \Omega }=0.$$

We examine the degeneracy of this *L* in accordance with the eigenvalues $0=\mu_{1}<\mu_{2}\leq \ldots \to \infty$ and the eigenfunctions *ϕ*_*j*_ in $\|\phi_{j}{\|}_{2}=1$ for j=1,2,…. Where *ϕ* is the eigenfunction of −Δ with Neumann boundary conditions.

First, for $\mu_{1}=0$, it holds that $\phi_{1}=\text{constant}$ and this condition is equivalent to

$$\delta +Da(u_{*})+Da^{\prime }(u_{*})u_{*}+\frac{a^{\prime }(u_{*})\xi }{\tau |\Omega |}\int_{\Omega }u_{*}\;dx+\frac{a(u_{*})\xi }{\tau |\Omega |}=\frac{a^{\prime }(u_{*})}{\tau }\lambda ,$$

although the possible bifurcated object is spatially homogeneous. Second, for *μ*_*j*_, *j* ≥ 2, it holds that $\int_{\Omega }\phi_{j}=0$, and the above degeneracy condition is reduced to

$$D\mu_{j}+\delta +Da(u_{*})+Da^{\prime }(u_{*})u_{*}+\frac{a^{\prime }(u_{*})\xi }{\tau |\Omega |}\int_{\Omega }u_{*}\;dx=\frac{a^{\prime }(u_{*})}{\tau }\lambda .$$

Then, there is a chance of a spatially inhomogeneous bifurcation.

From the above analysis, our main target is revealed. We want to prove that when *D* ≫ 1 is the case, in relation to *λ*, the solution (*u*, *v*) is asymptotically spatially homogeneous. The region that this holds cannot be the entire one because of the possible spatially inhomogeneous bifurcation of stationary states suggested above. Our result in the paper is the following theorem valid under the technical assumption

(34) $$N\leq 3,\;2|\xi |a_{1}<\tau^{3}(\mu_{2}D+\delta ).$$

Recall that *μ*_2_ > 0 is the second eigenvalue of −Δ under the Neumann boundary condition and *a*_1_ = *b*(*γ* + *k*_0_) is the upper bound of *a*(*u*) in (24). Note that ξ=1−τD>0 is not assumed in the following theorem and inequality (34) above and inequality (35) below are consistent if *δ* is sufficiently large.

Theorem 5 Assume (34). There exists a constant $\sigma =\sigma (b,\gamma ,k,k_{0},\tau )>0$ such that

(35) $$\sigma (1+\frac{\lambda^{2}}{D})\leq \delta$$

implies

(36) $$\underset{t\to \infty }{\lim}\|u(\cdot ,t)-ū(t),v(\cdot ,t)-\bar{v}(t){\|}_{\infty }=0$$

for the solution (u,v)=(u(x,t),v(x,t)) to (25) for (23), where

$$ū(t)=\frac{1}{\left|\Omega \right|}\int_{\Omega }u(x,t)\;dx,\;\bar{v}(t)=\frac{1}{\left|\Omega \right|}\int_{\Omega }v(x,t)\;dx.$$

The *ω*‐limit set *ω*(*u*_0_, *v*_0_) defined by (8) satisfies

(37) $$(u_{*},v_{*})\in \omega (u_{0},v_{0})\subset F_{\lambda }$$

for some (*u*_∗_, *v*_∗_) ∈ *G*_*λ*_ and

(38) $$F_{\lambda }=\{({ũ}_{*},{\tilde{v}}_{*})\in {\bar{\text{R}}}_{+}^{2}|{ũ}_{*}+\tau {\tilde{v}}_{*}=\lambda \}.$$

So far, spatially homogenization in reaction–diffusion system under the Neumann boundary condition with fast diffusion has been studied in several contexts. In Conway et al.,^14^ this property is shown under the presence of the invariant region. Even without this property, the local theory^15^ assures that spatially homogeneous compact attractor is also an attractor of the corresponding spatially inhomogeneous system. Both results are significantly based on the theory of dynamical systems. In these works, the exponential convergence rate to the spatially homogeneous part is assured, which our method did not reach. Theorem 5, however, assures that any solution exhibits asymptotic spatial homogeneity under the cost of large *δ* in this fourth model.

Since wave‐propagation phenomena are reported in numerical simulations,^11^, ^13^ we can suspect some dynamics inside *ω*(*u*_0_, *v*_0_) for the general case. In accordance with the conclusion (37), there is a possibility of *ω*(*u*_0_, *v*_0_) ≠ {(*u*_∗_(*λ*), *v*_∗_(*λ*))}, as this *ω*‐limit set contains nontrivial spatially homogeneous orbit of (25). See the final remark of the present paper. Concluding the present section, we refer to Henry^16^ for fundamental concepts on the dynamical systems, *ω*‐limit sets and LaSalle's principle used below.

## 4 PROOF OF THEOREM 5

Using w=Du+v, we transform (25) into

(39) $$\begin{array}{ccc} & u_{t}-D\Delta u+\theta (u)=a(u)w, & \\ & \tau w_{t}+\xi u_{t}=\Delta w\;\;\;\;\;\text{in}\;\Omega \times (0,T)\\ & \frac{\partial }{\partial \nu }(u,w)=0\;\;\;\;\;\text{on}\;\partial \Omega \times (0,T)\\ & {\left.\left(u,w\right)\right|}_{t=0}=(u_{0}(x),w_{0}(x))\;\;\text{in}\;\Omega , \end{array}$$

where

$$w_{0}=Du_{0}+v_{0},\;\xi =1-\tau D,\;\theta (u)=bu+Da(u)u.$$

Lemma 6 The solution (*u*, *w*) to (39) satisfies the estimate:

(40) $$\int_{0}^{T}(w-\bar{w},\tau w+\xi u-\lambda )\;dt\leq C_{3},$$

where (· , ·) is the usual inner product in *L*^2^(Ω) and

$$\bar{w}=\frac{1}{\left|\Omega \right|}\int_{\Omega }w\;dx.$$

Recalling

$$\tau w+\xi u=u+\tau v,\;\frac{1}{\left|\Omega \right|}\int_{\Omega }u+\tau v\;dx=\lambda ,$$

we obtain

(41) $${\left(\tau w+\xi u-\lambda \right)}_{t}-\Delta (w-\bar{w})=0,\;{\left.\frac{\partial w}{\partial \nu }\right|}_{\partial \Omega }=0$$

by (39). By

$$\int_{\Omega }\tau w+\xi u-\lambda \;dx=0,$$

it holds that

$${\left(-\Delta \right)}^{-1}{\left(\tau w+\xi u-\lambda \right)}_{t}+(w-\bar{w})=0,$$

where (− Δ)^−1^*f* = *z* denotes

$$-\Delta z=f\;\text{in}\;\Omega ,\;{\left.\frac{\partial z}{\partial \nu }\right|}_{\partial \Omega }=0,\;\int_{\Omega }z\;dx=0$$

for *f* satisfying

$$\int_{\Omega }f\;dx=0.$$

Hence, there arises

$$\left({\left(-\Delta \right)}^{-1}{\left(\tau w+\xi u-\lambda \right)}_{t},\tau w+\xi u-\lambda \right)+\left(w-\bar{w},\tau w+\xi u-\lambda \right)=0.$$

(− Δ)^−1^ is a bounded self‐adjoint operator in $E=\{f\in L^{2}(\Omega )|\int_{\Omega }f\;dx=0\}$, and therefore, it holds that

$$\frac{1}{2}\frac{d}{dt}\left({\left(-\Delta \right)}^{-1}(\tau w+\xi u-\lambda ),\tau w+\xi u-\lambda \right)+\left(w-\bar{w},\tau w+\xi u-\lambda \right)=0.$$

Then we obtain (40) because of the positivity of (− Δ)^−1^. □

Since

$$\lambda =\tau \bar{w}+\xi ū,\;ū=\frac{1}{\left|\Omega \right|}\int_{\Omega }u\;dx,$$

we get

(42) $$\tau \int_{0}^{T}\|w-\bar{w}{\|}_{2}^{2}\;dt\leq |\xi |{\left(\int_{0}^{T}\|w-\bar{w}{\|}_{2}^{2}\;dt\right)}^{\frac{1}{2}}{\left(\int_{0}^{T}\|u-ū{\|}_{2}^{2}\;dt\right)}^{\frac{1}{2}}+C_{3}$$

by (40). Here, a result on the boundedness of *v* = *w* − *Du* follows from the second equation of (25).

In the following lemma, *C*_4_ is a constant independent of *v*_0_. Actually, we use the semigroup estimate to bound ‖*v*(· , *t*)‖_2_ above by ‖*v*_0_‖_1_ + ‖*u*_0_‖_1_, where the conditions *t* ≥ 1 and *N* ≤ 3 are required.

Lemma 7 If *N* ≤ 3, then

(43) $$\|v(\cdot ,t){\|}_{2}\leq C_{4}\lambda ,\;t\geq 1.$$

By

$$\tau v_{t}\leq \Delta v-a_{0}v+\delta u,\;{\left.\frac{\partial v}{\partial \nu }\right|}_{\partial \Omega }=0,\;{\left.v\right|}_{t=0}=v_{0}(x),$$

we argue as in Latos et al.,^17^ recalling *a*_0_ = *bγ* > 0 in (24). First, we apply the comparison theorem to deduce

(44) $$\|v(\cdot ,t){\|}_{2}\leq \|e^{\tau^{-1}(\Delta -a_{0})t}v_{0}{\|}_{2}+\tau^{-1}\delta \int_{0}^{t}\|e^{\tau^{-1}(\Delta -a_{0})(t-s)}u(s){\|}_{2}\;ds,$$

where Δ is provided with the homogeneous Neumann boundary condition.

Second, the semigroup estimate^18^

$$\|e^{t\Delta }z{\|}_{p}\leq C_{5}\max\{1,t^{-\frac{N}{2}(\frac{1}{q}-\frac{1}{p})}\}\|z{\|}_{q},\;1\leq q\leq p\leq \infty$$

is applied to the right‐hand side of (44). It follows that

$$\begin{array}{ccc} \|v(\cdot ,t){\|}_{2} & \leq C_{5}\{\|v_{0}{\|}_{1}+\tau^{-1}\delta \int_{0}^{t}e^{-\tau^{-1}a_{0}(t-s)}{\left(t-s\right)}^{-\frac{N}{4}}\|u(s){\|}_{1}\;ds & \\ & \leq \lambda C_{6}\left\{1+\tau^{-1}\delta \int_{0}^{\infty }e^{-\tau^{-1}a_{0}t}t^{-\frac{N}{4}}\;dt\right\}=C_{7}\lambda , \end{array}$$

provided that *t* ≥ 1 and *N* ≤ 3. □

Recall *a*_1_ = *b*(*γ* + *k*_0_) in (24).

Lemma 8 If

(45) $$a_{1}(1+\frac{1}{2\tau })+\frac{2}{D}{\left(\mu_{2}^{-\frac{1}{2}}b\gamma \alpha (k)C_{4}\lambda \right)}^{2}\leq \frac{\delta }{2},$$

it holds that

(46) $$\int_{0}^{T}\|u-ū{\|}_{2}^{2}\;dt\leq \frac{a_{1}}{\tau (\mu_{2}D+\delta )}\int_{0}^{T}\|w-\bar{w}{\|}_{2}^{2}\;dt+C_{8}.$$

In this proof, we use the notations

$$\|z{\|}_{2}^{2}=\frac{1}{\left|\Omega \right|}\int_{\Omega }z^{2}\;dx,\;(z,\zeta )=\frac{1}{\left|\Omega \right|}\int_{\Omega }z\cdot \zeta \;dx,\;\bar{z}=\frac{1}{\left|\Omega \right|}\int_{\Omega }z\;dx.$$

Then it follows that

$$\;\|z-\bar{z}{\|}_{2}^{2}=\|z{\|}_{2}^{2}-{\bar{z}}^{2},\;(z,\zeta -\bar{\zeta })=(z-\bar{z},\zeta -\bar{\zeta }).$$

We begin by integrating over Ω the first equation of (25) to get

$${ū}_{t}+\delta ū=\bar{a(u)v},$$

and then multiplying with ū,

$$\frac{1}{2}\frac{d}{dt}{ū}^{2}+\delta {ū}^{2}=\bar{a(u)v}\cdot ū.$$

Next, we test the first equation of (25) with *u*:

$$\frac{1}{2}\frac{d}{dt}\|u{\|}_{2}^{2}+D\|\nabla u{\|}_{2}^{2}+\delta \|u{\|}_{2}^{2}=(a(u)v,u).$$

Subtracting the last two relations above, we calculate

(47) $$\begin{array}{ccc} & \frac{1}{2}\frac{d}{dt}\|u-ū{\|}_{2}^{2}+D\|\nabla u{\|}_{2}^{2}+\delta \|u-ū{\|}_{2}^{2}=(a(u)v,u)-\bar{a(u)v}\cdot ū & \\ & \;=\left(a(u)v,u-ū\right)=(a(u)v-\bar{a(u)v},u-ū)\\ & \;=(a(u)(v-\bar{v}),u-ū)+(a(u)\bar{v}-\bar{a(u)v},u-ū). \end{array}$$

Moreover, we have

$$a(u)\bar{v}-\bar{a(u)v}=a(u)\frac{1}{\left|\Omega \right|}\int_{\Omega }v\;dy-\frac{1}{\left|\Omega \right|}\int_{\Omega }a(u)v\;dy$$

or

$$a(u(x,t))\bar{v}-\bar{a(u)v}=\frac{1}{\left|\Omega \right|}\int_{\Omega }\left[a(u(x,t))-a(u(y,t))\right]v(y,t)\;dy,$$

which implies

$$\|a(u)\bar{v}-\bar{a(u)v}{\|}_{2}^{2}\leq \|v{\|}_{2}^{2}\cdot \frac{1}{|\Omega {|}^{2}}\iint_{\Omega \times \Omega }{\left[a(u(x,t))-a(u(y,t))\right]}^{2}\;dxdy.$$

Here, the Poincaré–Wirtinger inequality implies

(48) $$\begin{array}{ccc} & \frac{1}{|\Omega {|}^{2}}\iint_{\Omega \times \Omega }|z(x)-z(y){|}^{2}\;dxdy & \\ & \;\leq 2\cdot \frac{1}{|\Omega {|}^{2}}\iint_{\Omega \times \Omega }|z(x)-\bar{z}{|}^{2}+|z(y)-\bar{z}{|}^{2}\;dxdy\\ & \;=4\|z-\bar{z}{\|}^{2}\leq 4\mu_{2}^{-1}\|\nabla z{\|}_{2}^{2}. \end{array}$$

Therefore, (47) with the help of (48) blue,

$$\begin{array}{ccc} & \frac{1}{2}\frac{d}{dt}\|u-ū{\|}_{2}^{2}+D\|\nabla u{\|}_{2}^{2}+\delta \|u-ū{\|}_{2}^{2} & \\ & \;\leq a_{1}\|u-ū{\|}_{2}\cdot \|v-\bar{v}{\|}_{2}+2\mu_{2}^{-\frac{1}{2}}\|v{\|}_{2}\|\nabla a(u){\|}_{2}\cdot \|u-ū{\|}_{2}\\ & \;\leq a_{1}\left\{D\|u-ū{\|}_{2}^{2}+\|w-\bar{w}{\|}_{2}\cdot \|u-ū{\|}_{2}\right\}+2b\mu_{2}^{-\frac{1}{2}}\gamma \alpha (k)\|v{\|}_{2}\|\nabla u{\|}_{2}\|u-ū{\|}_{2}\\ & \;\leq a_{1}D\|u-ū{\|}_{2}^{2}+\frac{a_{1}}{2\tau }\|w-\bar{w}{\|}_{2}^{2}+\frac{a_{1}}{2\tau }\|u-ū{\|}_{2}^{2}+\frac{D}{2}\|\nabla u{\|}_{2}^{2}\\ & \;+\frac{1}{2D}{\left(2\mu_{2}^{-\frac{1}{2}}b\gamma \alpha (k)C_{4}\lambda \right)}^{2}\|u-ū{\|}_{2}^{2} \end{array}$$

by (24), and hence,

$$\begin{array}{ccc} & \frac{1}{2}\frac{d}{dt}\|u-ū{\|}_{2}^{2}+(\frac{\mu_{2}D}{2}+\delta )\|u-ū{\|}_{2}^{2} & \\ & \;\leq \left[a_{1}(1+\frac{1}{2\tau })+\frac{2}{D}{\left(\mu_{2}^{-\frac{1}{2}}b\gamma \alpha (k)C_{4}\lambda \right)}^{2}\right]\|u-ū{\|}_{2}^{2}+\frac{a_{1}}{2\tau }\|w-\bar{w}{\|}_{2}^{2} \end{array}$$

by *a*_1_ = *b*(*γ* + *k*_0_).

Thus, we conclude

$$\frac{d}{dt}\|u-ū{\|}_{2}^{2}+(\mu_{2}D+\delta )\|u-ū{\|}_{2}^{2}\leq \frac{a_{1}}{\tau }\|w-\bar{w}{\|}_{2}^{2}$$

from (45) and then obtain (46). □

Inequality (45) arises if we have (35) for $\sigma =\sigma (b,\gamma ,k,k_{0},\tau )>0$ sufficiently large. Then we obtain the following lemma, recalling (8) and (38).

Lemma 9 Assume (34) and inequality (35) for *σ* > 0 as above. Then it holds that (36) and *ω*(*u*_0_, *v*_0_) ⊂ *F*_*λ*_.

From (42) and (46), we get

$$\begin{array}{ccc} & \tau \int_{0}^{T}\|w-\bar{w}{\|}_{2}^{2}\;dt & \\ & \;\leq |\xi |{\left(\int_{0}^{T}\|w-\bar{w}{\|}_{2}^{2}\;dt\right)}^{\frac{1}{2}}\cdot {\left(\frac{a_{1}}{\tau (\mu_{2}D+\delta )}\int_{0}^{T}\|w-\bar{w}{\|}_{2}^{2}\;dt+C_{8}\right)}^{\frac{1}{2}}+C_{3}. \end{array}$$

Then (34) blue

$$\int_{0}^{T}\|w-\bar{w}{\|}_{2}^{2}\;dt\leq C_{9},$$

and hence,

(49) $$\int_{0}^{\infty }\|w-\bar{w}{\|}_{2}^{2}\;dt<+\infty .$$

From the parabolic regularity and uniformly boundedness of (u,v)=(u(x,t),v(x,t)), the mapping $t\to \|w-\bar{w}{\|}_{2}^{2}$ is uniformly continuous, and therefore, we have

$$\underset{t\to \infty }{\lim}\|w-\bar{w}{\|}_{2}^{2}=0$$

by (49). Then, again using the parabolic regularity, we get

$$\underset{t\to \infty }{\lim}\|w-\bar{w}{\|}_{\infty }=0.$$

Since (49) implies also

$$\int_{0}^{\infty }\|u-ū{\|}_{2}^{2}\;dt<+\infty$$

by (46), it holds that

$$\underset{t\to \infty }{\lim}\|u-ū{\|}_{\infty }=0$$

similarly. We thus obtain (36).

Given $\left({\hat{u}}_{*},{\hat{v}}_{*})\in \omega (u_{0},v_{0}\right)$, finally, we have *t*_*k*_ → *∞* such that

$$\underset{k\to \infty }{\lim}\|u(t_{k})-{\hat{u}}_{*},v(t_{k})-{\hat{v}}_{*}{\|}_{\infty }=0.$$

Then it holds that

$$\lambda =ū(t_{k})+\tau \bar{v}(t_{k})\;\to \;{\hat{u}}_{*}+\tau {\hat{v}}_{*}$$

and hence $({\hat{u}}_{*},{\hat{v}}_{*})\in F_{\lambda }$. □

Now we study the spatially homogeneous part of (25).

Lemma 10 Take $\left({ũ}_{*},{\tilde{v}}_{*})\in {\bar{\text{R}}}_{+}^{2}\backslash \{(0,0)\right\}$ and let (U,V)=(U(t),V(t)) be the solution to (25) for $\left(u_{0},v_{0})=({ũ}_{*},{\tilde{v}}_{*}\right)$:

(50) $$\frac{dU}{dt}=f(U,V),\;\tau \frac{dV}{dt}=-f(U,V),\;{\left.\left(U,V\right)\right|}_{t=0}=({ũ}_{*},{\tilde{v}}_{*}).$$

Put $\lambda ={ũ}_{*}+\tau {\tilde{v}}_{*}>0$ and define *G*_*λ*_ as in Proposition 1. Then it holds that

(51) $$\emptyset \neq \omega ({ũ}_{*},{\tilde{v}}_{*})\in G_{\lambda }.$$

First, we have

$$\frac{d}{dt}(U+\tau V)=0,$$

and hence,

(52) U+τV=λ.

Then (50) is reduced to the single system,

(53) $$\frac{dU}{dt}=-\delta U+a(U)\tau^{-1}(\lambda -U),\;{\left.U\right|}_{t=0}={ũ}_{*}\geq 0$$

by (22) and (23). This system is a spatially homogeneous part of (25), and therefore, there is a global‐in‐time uniformly bounded orbit O={U(t)} in ${\bar{\text{R}}}_{+}$.

Finding *G*(*U*) such that

$$G^{\prime }(U)=-\delta U+a(U)\tau^{-1}(\lambda -U),$$

we obtain

$$\frac{d}{dt}G(U)=G^{\prime }(U)\frac{dU}{dt}={\left[G^{\prime }(U)\right]}^{2}\geq 0.$$

This −*G*(*U*) is a Lyapunov functional, and therefore, there is

$$\underset{t\to \infty }{\lim}G(U(t))=G_{\infty }$$

from the compactness of O.

The *ω*‐limit set for (53) is defined by

$$\omega ({ũ}_{*})=\{{\hat{u}}_{*}|\exists t_{k}\to \infty \;\text{such that}\;\underset{k\to \infty }{\lim}U(t_{k})={\hat{u}}_{*}\}.$$

It is invariant under the flow defined by (53). Hence, given ${\hat{u}}_{*}\in \omega ({ũ}_{*})$, it holds that

$$Ũ(t)\in \omega ({ũ}_{*}),\;t\geq 0$$

for the solution Ũ=Ũ(t) to (53) with the initial value ${ũ}_{*}$ replaced by ${\hat{u}}_{*}$. It also holds that

$$G({\hat{u}}_{*})=G_{\infty },\;\forall {\hat{u}}_{*}\in \omega ({ũ}_{*}),$$

and hence, *G′*(*U*(*t*)) ≡ 0 (LaSalle's principle).

This property means that (*U*(*t*), *V*(*t*)) with *V*(*t*) defined by (52) is a spatially homogeneous solution in Proposition 1, which implies

$$\omega ({ũ}_{*})=\{u_{*}(\lambda )\}.$$

Thus we obtain $\lim_{t\to \infty }U(t)=u_{*}(\lambda )$, and hence, $\lim_{t\to \infty }V(t)=v_{*}(\lambda )$ again by (52). □

Finally, we combine all the above results to prove our main theorem:

Proof of Theorem 5 It suffices to show the existence of (*u*_∗_, *v*_∗_) ∈ *G*_*λ*_ such that (*u*_∗_, *v*_∗_) ∈ *ω*(*u*_0_, *v*_0_).

Given $({ũ}_{*},{\tilde{v}}_{*})\in \omega (u_{0},v_{0})\subset F_{\lambda }$, let (*U*, *V*) be the solution to (25) with the initial value (*u*_0_, *v*_0_) replaced by $\left({ũ}_{*},{\tilde{v}}_{*}\right)$. This solution (U,V)=(U(t),V(t)) is spatially homogeneous as in (50), and hence, it follows that (51). Then we obtain $\tilde{\omega }({ũ}_{*},{\tilde{v}}_{*})\subset \omega (u_{0},v_{0})$ from the invariance of the *ω*‐limit set under the flow of (25). Then the result follows. □

Remark 4 If $\exists ({ũ}_{*},{\tilde{v}}_{*})\in \omega (u_{0},v_{0})\backslash G_{\lambda }$ is the case, there arises {(*U*(*t*), *V*(*t*))} ⊂ *ω*(*u*_0_, *v*_0_) for U=U(t) and V=V(t) defined by (53) and (52).

## CONFLICT OF INTEREST

This work does not have any conflicts of interest.
